# Characterization of the acute inflammatory profile and resolution of airway inflammation after *Igf1r*-gene targeting in a murine model of HDM-induced asthma

**DOI:** 10.1371/journal.pone.0190159

**Published:** 2017-12-22

**Authors:** Sergio Piñeiro-Hermida, Elvira Alfaro-Arnedo, Joshua A. Gregory, Raquel Torrens, Carlos Ruíz-Martínez, Mikael Adner, Icíar P. López, José G. Pichel

**Affiliations:** 1 Lung Cancer and Respiratory Diseases Unit, Centro de Investigación Biomédica de la Rioja (CIBIR), Fundación Rioja Salud, Logroño, Spain; 2 Unit of Experimental Asthma and Allergy Research, Karolinska Institutet, Institute of Environmental Medicine (IMM), Stockholm, Sweden; 3 Pneumology Service, Hospital San Pedro, Logroño, Spain; Centre National de la Recherche Scientifique, FRANCE

## Abstract

Asthma is a chronic inflammatory disease characterized by bronchial hyperresponsiveness, mucus overproduction and airway remodeling. Notably, we have recently demonstrated that insulin-like growth factor 1 receptor (IGF1R) deficiency in mice attenuates airway hyperresponsiveness and mucus secretion after chronic house dust mite (HDM) exposure. On this basis, inbred C57BL/6 and *Igf1r*-deficient mice were given HDM extract to study the acute inflammatory profile and implication of *Igf1r* in acute asthma pathobiology. Additionally, *Igf1r*-deficiency was therapeutically induced in mice to evaluate the resolution of HDM-induced inflammation. Acute HDM exposure in inbred C57BL/6 mice led to a progressive increase in inflammation, airway remodeling and associated molecular indicators. Preventively-induced *Igf1r*-deficiency showed reduced neutrophil and eosinophil numbers in BALF and bone marrow, a significant reduction of airway remodeling and decreased levels of related markers. In addition, therapeutic targeting of *Igf1r* promoted the resolution of HDM-induced-inflammation. Our results demonstrate for the first time that *Igf1r* is important in acute asthma pathobiology and resolution of HDM-induced inflammation. Thus, IGF1R is suggested to be a promising candidate for future therapeutic approaches for the treatment and prevention of asthma.

## Introduction

Asthma is a chronic inflammatory disease characterized by airway hyperresponsiveness (AHR), mucus overproduction and airway wall remodeling [1]. House dust mite (HDM)-derived allergens and specifically those arising from the species *Dermatophagoides pteronyssinus* are the most important source of mite-related allergens in asthmatic patients [2]. In response to allergen stimulation the airway epithelium secretes fluids, antimicrobial proteins, and mucins, which together with club cells represent a major component of the immunomodulatory barrier of the airway epithelium [3].

The insulin-like growth factor 1 receptor (IGF1R) is a ubiquitously expressed tyrosine kinase and a central member of the IGF axis. The IGF axis is comprised of two major ligands (IGF1 and IGF2), different receptors and regulatory proteins such as IGF-binding proteins (IGFBPs), acting together to control a number of essential cellular functions including proliferation, differentiation, survival, adhesion and migration [4]. Although little is known about the role of IGFs in human asthma, IGF1 and IGFBP3 were suggested to be involved in allergic airway inflammation and remodeling [5,6]. Additionally, IGF1R was found to be upregulated in BAL cells of asthmatic patients [7]. In mice, IGF1 was reported to be a relevant mediator of allergic airway inflammation and remodeling, and that IGFBP3 blocks the specific physiological consequences of this pathology [8–10]. Notably, we have recently reported that IGF1R plays an important role in initiation of the inflammatory response in mice, and the importance of IGF1R in the pathogenesis of murine asthma, mediating both AHR and mucus secretion after chronic HDM exposure [11,12]. Thus, we aimed to further investigate the involvement of IGF1R in allergic airway inflammation. For this purpose, *Igf1r* deficiency was preventively or therapeutically induced in mice to evaluate the implication of *Igf1r* in acute asthma pathobiology and resolution of airway inflammation following HDM exposure. The main finding of this study is that therapeutic targeted deletion of *Igf1r* resolves HDM-induced inflammation in mice. We assert that IGF1R is suggested to be a promising candidate for future therapeutic approaches for the treatment and prevention of asthma.

## Materials and methods

### Ethics approval

All experiments and animal procedures conducted were carried out in accordance with the guidelines of the European Communities Council Directive (86/609/EEC) and were revised and approved by the CIBIR Bioethics Committee (refs. JGP02_1 and JGP02_2).

### HDM sensitization protocols and preventive and therapeutic targeting of *Igf1r*

Eight- to 10-week-old (W8-10) female mice were intranasally challenged with consecutive doses of 40 μg of HDM extract (Greer Laboratories Inc, Lenoir, NC, USA) in 20 μl of PBS (2 mg/ml) or equal volume of PBS under light isoflurane anesthesia. Females were used due to their reported higher susceptibility to allergic airway inflammation [13]. Three different protocols of HDM sensitization were used: i) inbred C57Bl/6 mice were given seven doses of HDM extract or PBS and BALF and lungs were collected 24 h after the last exposure on days (D) 3, D5 or D7 (acute HDM protocol); ii) *Igf1r*^*fl/fl*^ (controls) and *UBC-CreERT2; Igf1r*^*fl/fl*^ mice were treated with tamoxifen (TMX) for five consecutive days at four weeks of age to induce a postnatal *Igf1r* gene deletion in *UBC-CreERT2; Igf1r*^*fl/fl*^ mice [14]. After TMX treatment, *UBC-CreERT2; Igf1r*^*Δ/Δ*^ (*CreERT2*) and *Igf1r*^*fl/fl*^ mice were administered with seven doses of HDM extract or PBS and bone marrow, serum, BALF and lungs were harvested 24 h after last dose on D7 (prophylactic protocol); and iii) *Igf1r*^*fl/fl*^ and *UBC-CreERT2; Igf1r*^*fl/fl*^ mice were challenged with seven (first set of animals non-treated with TMX and sacrificed at D7) or fourteen doses of HDM extract or PBS (second set of animals receiving five consecutive intraperitoneal TMX injections between D7 and D11 to induce *Igf1r* deletion in *UBC-CreERT2; Igf1r*^*fl/fl*^ mice, to generate *CreERT2* mice), followed by serum, BALF and lung tissue collection 24 h after the last exposure (therapeutic protocol). *UBC-CreERT2; Igf1r*^*fl/fl*^ double transgenic mice were in a C57BL/6-enriched (at least six generation backcrosses to C57BL/6 strain) mixed genetic background. All animals were bred and maintained under specific pathogen-free conditions at CIBIR animal facilities.

### Tissue collection and preparation

Twenty-four hours after the final HDM exposure, animals were euthanized by intraperitoneal injection of 10 μl/g of a ketamine-xylazine anesthetic combination in saline (300:30 mg/kg respectively). Immediately, lungs were lavaged twice with 0.8 ml cold PBS to obtain the bronchoalveolar lavage fluid (BALF). Blood was collected by cardiac puncture, and serum was obtained and stored at -80°C until further use. Following lung dissection, the left lung was fixed in 4% formaldehyde and embedded in paraffin for histopathology. Right lobes were separated and snap-frozen in liquid nitrogen for quantitative real-time PCR (qRT-PCR) and ELISA analyses. Additionally, femoral bone marrow was isolated as previously described [11]. Three different sets of mice were used respectively for BALF, histology/molecular analyses and bone marrow cell counts.

### Quantification of BALF and bone marrow

Total cell number was counted and expressed as cells/ml BALF or bone marrow, and differential cell counts were performed on May-Grümbald/Giemsa (Sigma-Aldrich, St. Louis, MO, USA)-stained cytospins, counting a minimum of 300 cells per slide or five fields per slide in BALF and bone marrow cytospins, respectively. Determination of differential cell counts was performed using standard morphology criteria.

### Histopathological analysis

Paraffin embedded left lungs were cut into 3 μm sections, evaluating a minimum of four airways per animal. Hematoxylin and eosin (H&E) staining was performed for quantification of inflamed lung areas and airway thickness. Quantification of inflammation was conducted as previously described [11]. Airway thickness was assessed by means of three different measurements per airway. Periodic acid-Schiff (PAS) and Masson´s trichrome staining protocols served to visualize the degree of goblet cell hyperplasia and collagen deposition. Fiji open-source image processing software package v1.48r (http://fiji.sc) was used to quantify the area of inflammation, airway thickness, collagen content and epithelium length measurements.

### RNA isolation, qRT-PCR and ELISAS

RNA isolation and qRT-PCR was performed using inferior lung lobes, as previously described [12]. A full list of primer sets used is provided in S1 Table. Cytokines were analyzed in serum and tissue homogenate supernatants from middle lung lobes using mouse IL13 Duoset and IL10, IL33 and CCL11 Quantikine ELISA Kits (R&D systems, Minneapolis, MN, USA).

### Statistical analyses

Statistical analyses were carried out using SPSS Statistics Software v21 for windows (IBM, Armonk, NY, USA). Differences between experimental groups were evaluated for significance using the non-parametric Mann-Whitney U test or the Dunn-Sidak test for multiple comparisons. Results are shown as mean values ± standard error of the mean (SEM). For all analyses, a *p* value < 0.05 was considered statistically significant.

## Results

### Characterization of the murine acute allergic profile

**Fig 1:**
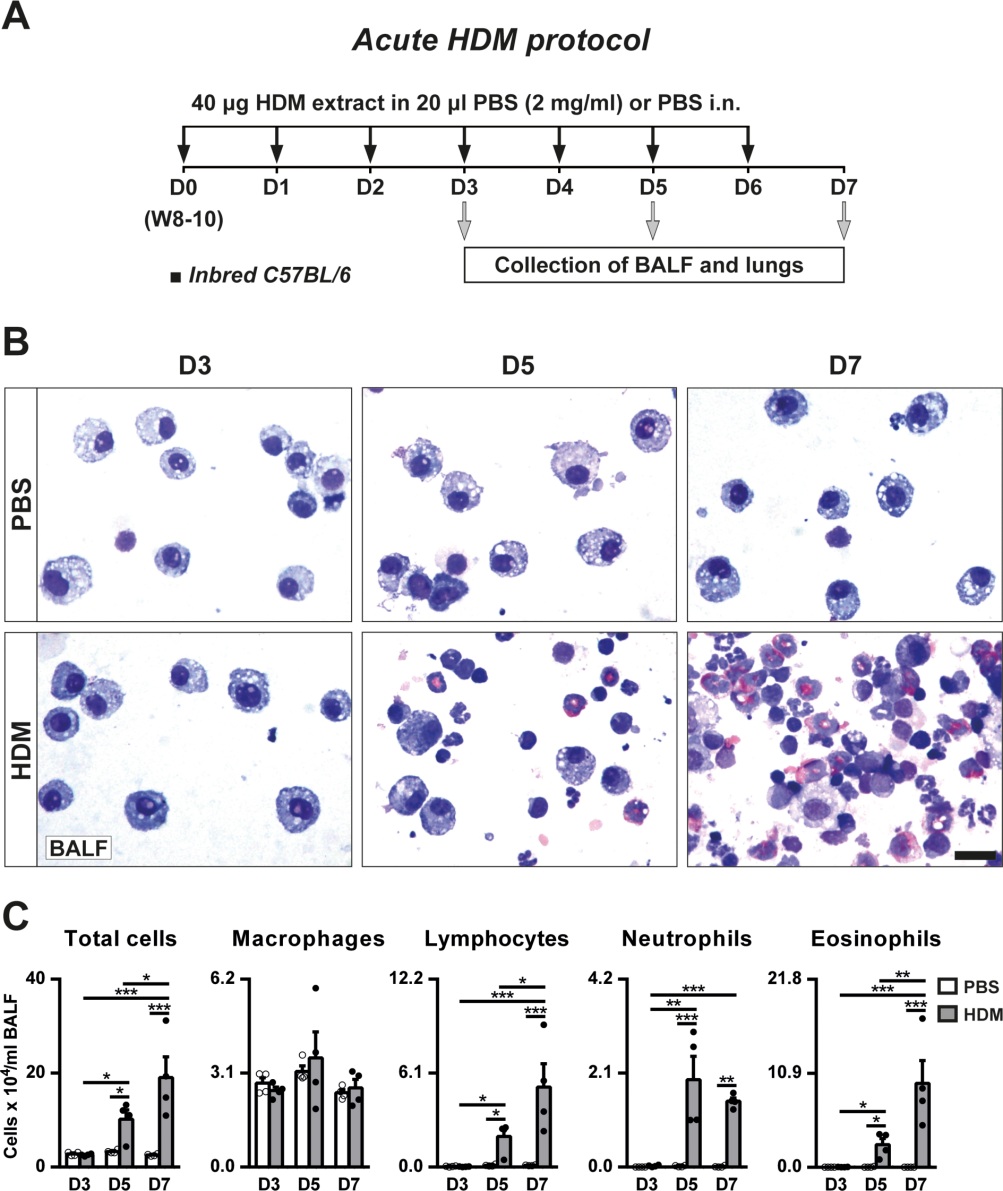
Protocol for acute HDM exposure and progressive accumulation of inflammatory cells in BALF of inbred C57BL/6 mice. (A) Eight- to 10-week-old (W8-10) inbred C57BL/6 female mice were intranasally challenged with daily consecutive doses of 40 μg of HDM extract in 20 μl of PBS (2 mg/ml) or equal volume of PBS. BALF and lungs were collected 24 h after the last exposure on days [D] 3, D5 or D7. (B-C) Representative images of BALF cytospin preparations (scale bar: 20 μm) and total and differential cell counts in BALF from PBS- or HDM-treated inbred C57BL/6 mice at D3, D5 and D7. Data are expressed as mean ± SEM (n = 4 animals per group). *p<0.05; **p<0.01; ***p<0.001 (Dunn-Sidak multiple comparison test). HDM, house dust mite; PBS, phosphate buffered saline.

Inbred C57BL/6 mice were subjected to an acute HDM exposure to study the progressive changes in BALF and lung (Fig 1A). Total cell counts were significantly increased at day D5 and D7, reaching the highest numbers at D7 (Fig 1B and 1C). Differential cell counts in BALF showed a marked increase in lymphocyte, neutrophil and eosinophil numbers at D5 and D7, whilst macrophage counts remained unchanged. Lymphocyte and eosinophil numbers were higher at D7 without changes in neutrophil counts between D5 and D7 (Fig 1B and 1C).

**Fig 2:**
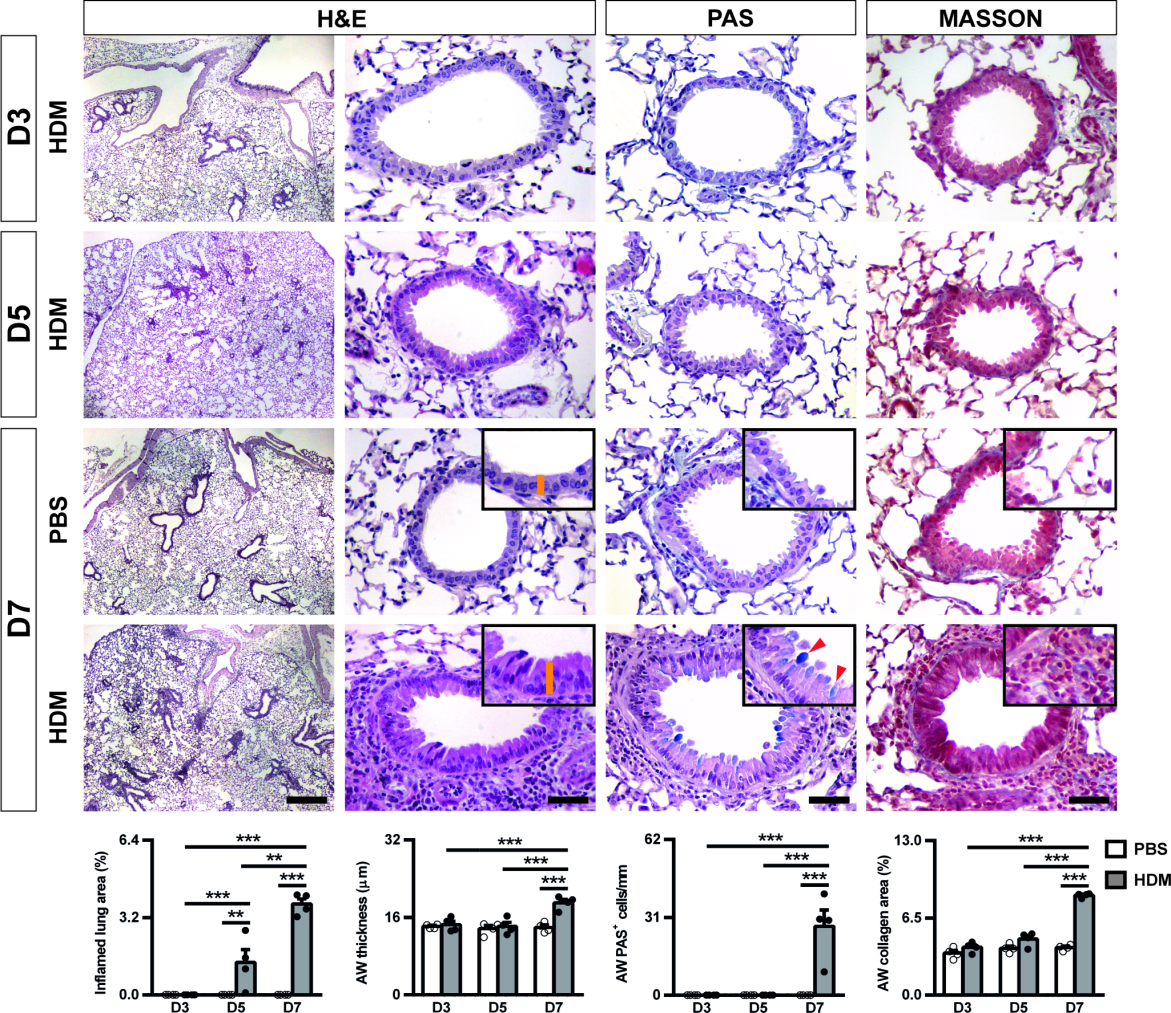
Progressive increase in airway inflammation and remodeling after acute HDM exposure in inbred C57BL/6 mice. Representative images of proximal airways showing inflamed lung areas (left) (scale bar: 0.5 mm), and airway thickness (orange bars in insets) (center left), mucus-producing cells per epithelium length (red arrowheads in insets) (center right) and collagen content (right) (scale bars: 50 μm) in PBS- or HDM-treated C57BL/6 mice at D3, D5 and D7. Bottom graphs represent quantification of the abovementioned parameters. Data are expressed as mean ± SEM (n = 4 animals per group). **p<0.01; ***p<0.001 (Dunn-Sidak multiple comparison test). H&E, Hematoxilin and eosin; PAS, Periodic Acid Schiff; AW, airway; HDM, house dust mite; PBS, phosphate buffered saline.

Inflamed lung area, airway thickness, number of PAS^+^ cells and collagen staining were only significantly changed at D7, with the exception of the inflamed lung area parameter which was increased at D5, although to a lesser degree than at D7 (Fig 2).

**Fig 3:**
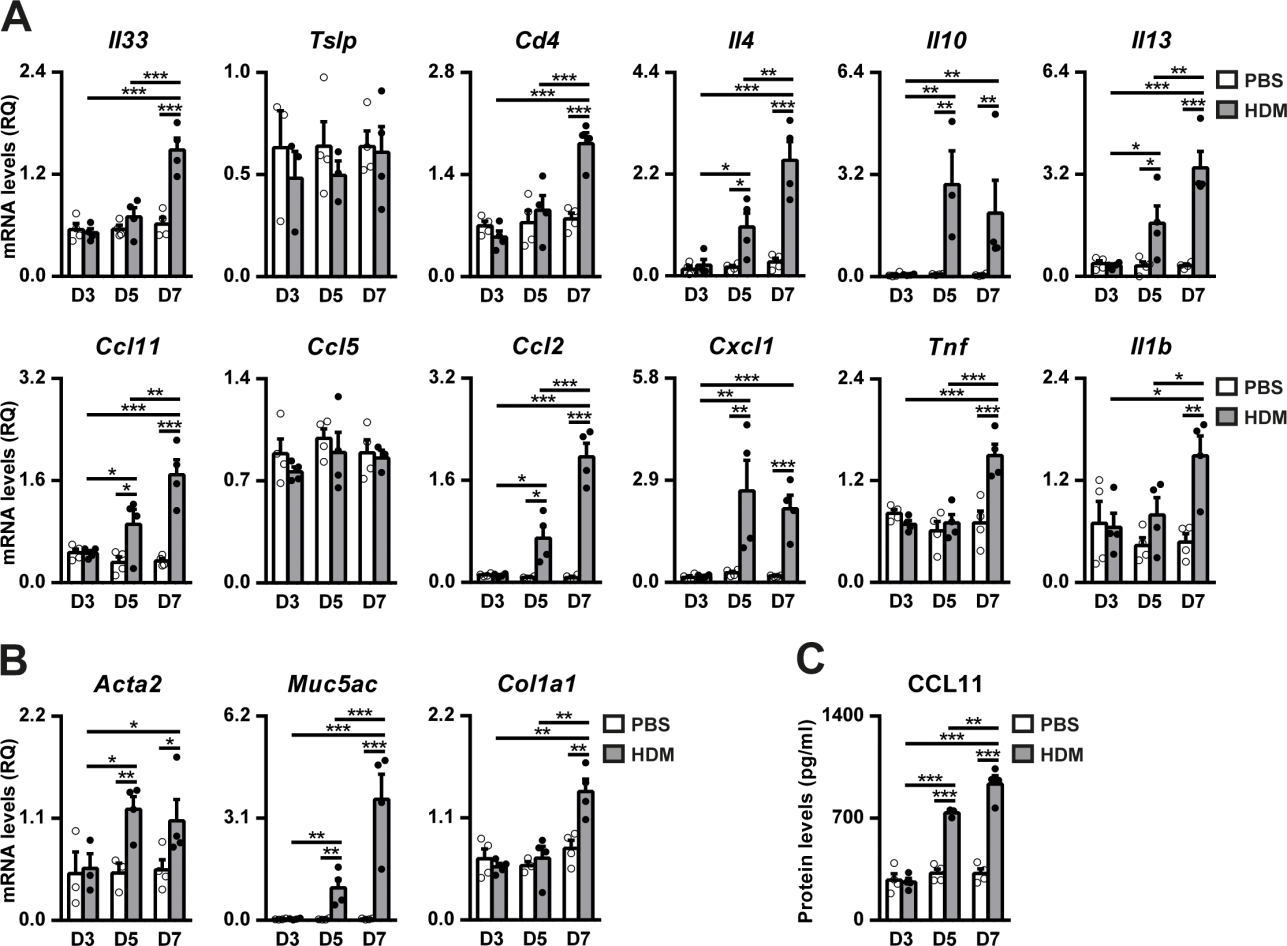
Expression of airway inflammation and remodeling markers after acute HDM treatment in inbred C57BL/6 mice. (A) Lung tissue mRNA expression levels of dendritic cell activators (*Il33* and *Tslp*), T-lymphocyte marker (*Cd4*), Th2 cytokines (*Il4*, *Il10* and *Il13*), eosinophil (*Ccl11* and *Ccl5*), macrophage (*Ccl2)* and neutrophil (*Cxcl1*) chemoattractants and Th1 cytokines (*Tnf* and *Il1b*); (B) bronchoconstriction (*Acta2*), goblet cell hyperplasia (*Muc5ac*) and collagen deposition (*Col1a1*) markers, and (C) CCL11 protein levels in lung homogenates in PBS- or HDM-treated inbred C57BL/6 mice at D3, D5 and D7. Data are expressed as mean ± SEM (n = 3–4 animals per group). *p<0.05; **p<0.01; ***p<0.001 (Dunn-Sidak multiple comparison test). HDM, house dust mite; PBS, phosphate buffered saline.

Lung mRNA expression analysis demonstrated a significant up-regulation of the allergic airway inflammation markers *Il33*, *Cd4*, *Il4*, *Il10*, *Il13*, *Ccl11*, *Ccl2*, *Cxcl1*, *Tnf* and *Il1b* in addition to the airway remodeling indicators *Acta2*, *Muc5ac* and *Col1a1*. *Tslp* and *Ccl5* expression was not found to be induced by HDM, and *Il25* expression was not able to be measured due to low to undetectable levels (Fig 3A and 3B). It should be noted that mRNA expression of *Il33*, *Cd4*, *Tnf*, *Il1b* and *Col1a1* markers was induced at D7 unlike all other markers which were already significantly increased at D5 but in general in a lesser extent than at D7. The increased *Ccl11* expression was validated by CCL11 protein levels (Fig 3C).

### Decreased HDM-induced neutrophilopoiesis and eosinophilopoiesis, and IL13, CCL11 and IgE serum levels after preventively-induced *Igf1r* deficiency

**Fig 4:**
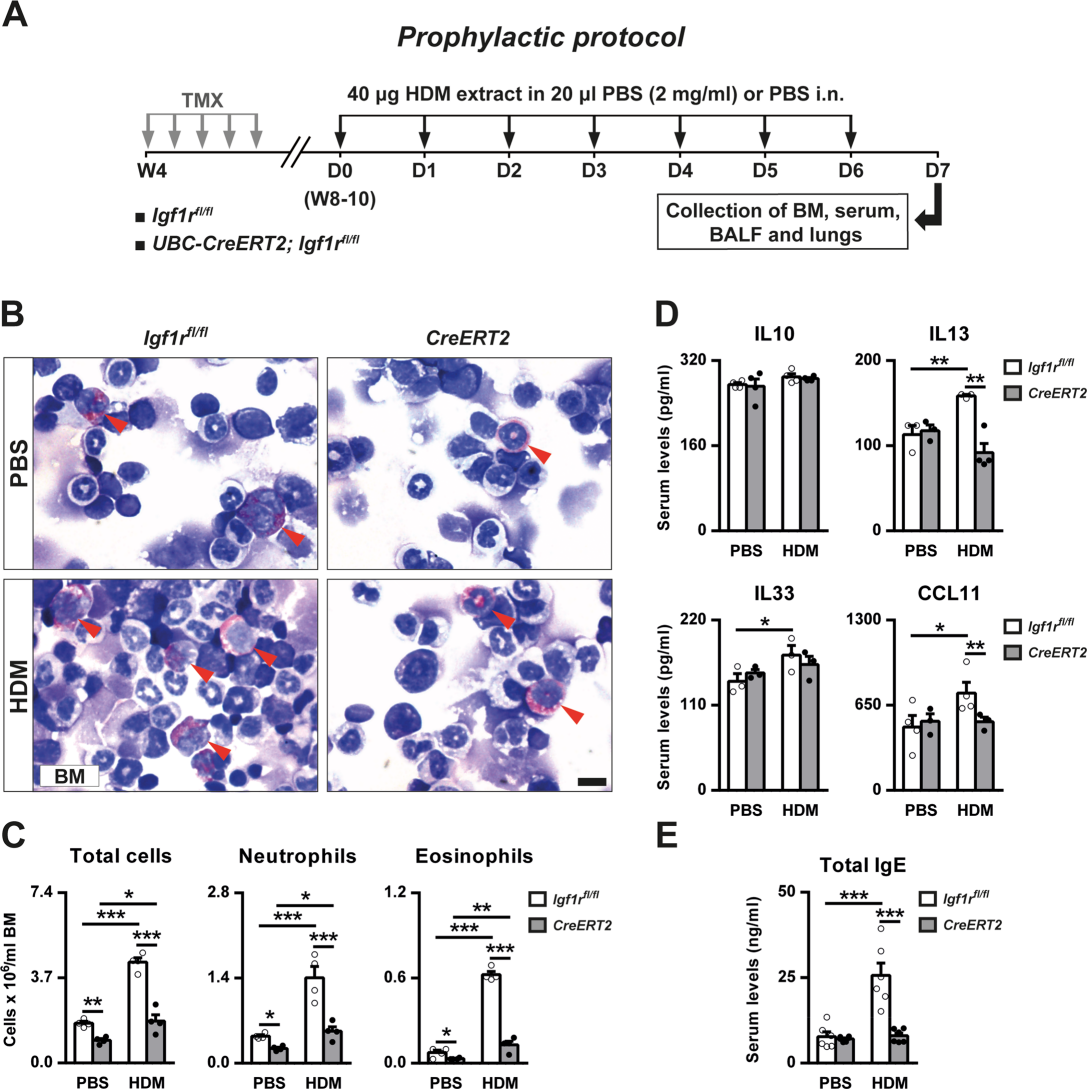
Protocol for prophylactic induction of *Igf1r* deficiency and HDM treatment, bone marrow cell counts and circulating levels of allergy-related markers. (A) *Igf1r*^*fl/fl*^ (controls) and *UBC-CreERT2; Igf1r*^*fl/fl*^ female mice were treated with tamoxifen (TMX) for five consecutive days at four weeks of age (W4) to induce a postnatal *Igf1r* gene deletion [14]. Then, eight- to 10-week-old (W8-10) *Igf1r*^*fl/fl*^ and *UBC-CreERT2; Igf1r*^*Δ/Δ*^ (*CreERT2*) female mice were intranasally challenged with seven daily consecutive doses of 40 μg of HDM extract in 20 μl of PBS (2 mg/ml) or equal volume of PBS. Bone marrow, serum, BALF and lungs were harvested 24 h after last dose on D7. (B-C) Representative images and total, neutrophil and eosinophil (red arrowheads) counts in bone marrow (BM) cytospin preparations (Scale bar: 10 μm; n = 4 animals per group) and (D-E) serum levels of IL10, IL13, IL33, CCL11 and IgE (n = 3–6 animals per group) in PBS- or HDM-exposed *Igf1r-*deficient vs. *Igf1*^*fl/fl*^ mice. Data are expressed as mean ± SEM. *p<0.05; **p<0.01; ***p<0.001 (Dunn-Sidak multiple comparison test). HDM, house dust mite; PBS, phosphate buffered saline.

Together, the results presented in the previous section indicate that D7 is an appropriate time point to study the acute allergic phenotype after HDM challenge. Thus, *Igf1r* deficiency was preventively-induced to study the implication of *Igf1r* in acute asthma pathobiology (Fig 4A).

Total and differential cell counts in bone marrow cytospins and measurement of serum levels of several cytokines and IgE were performed in *Igf1r*-deficient and *Igf1r*^*fl/fl*^ mice. Total, neutrophil and eosinophil counts in bone marrow were found to be diminished in HDM-treated *Igf1r*-depleted compared to *Igf1r*^*fl/fl*^ lungs. This phenomenon was also evident within PBS-treated groups. In spite of *Igf1r*^*fl/fl*^ mice showed increased neutrophil and eosinophil numbers after HDM treatment, *Igf1r*-deficient mice did not show such an increase (Fig 4B and 4C). Serum IL33 levels showed only a slight increase in HDM-exposed *Igf1r*^*fl/fl*^ mice. In addition, IL13, CCL11 and IgE levels were significantly increased in *Igf1r*^*fl/fl*^ mice after HDM treatment, whereas *Igf1r*-depleted mice exhibited normal values (Fig 4D and 4E).

### Preventively-induced *Igf1r* deficiency reduces inflammation and remodeling features

**Fig 5:**
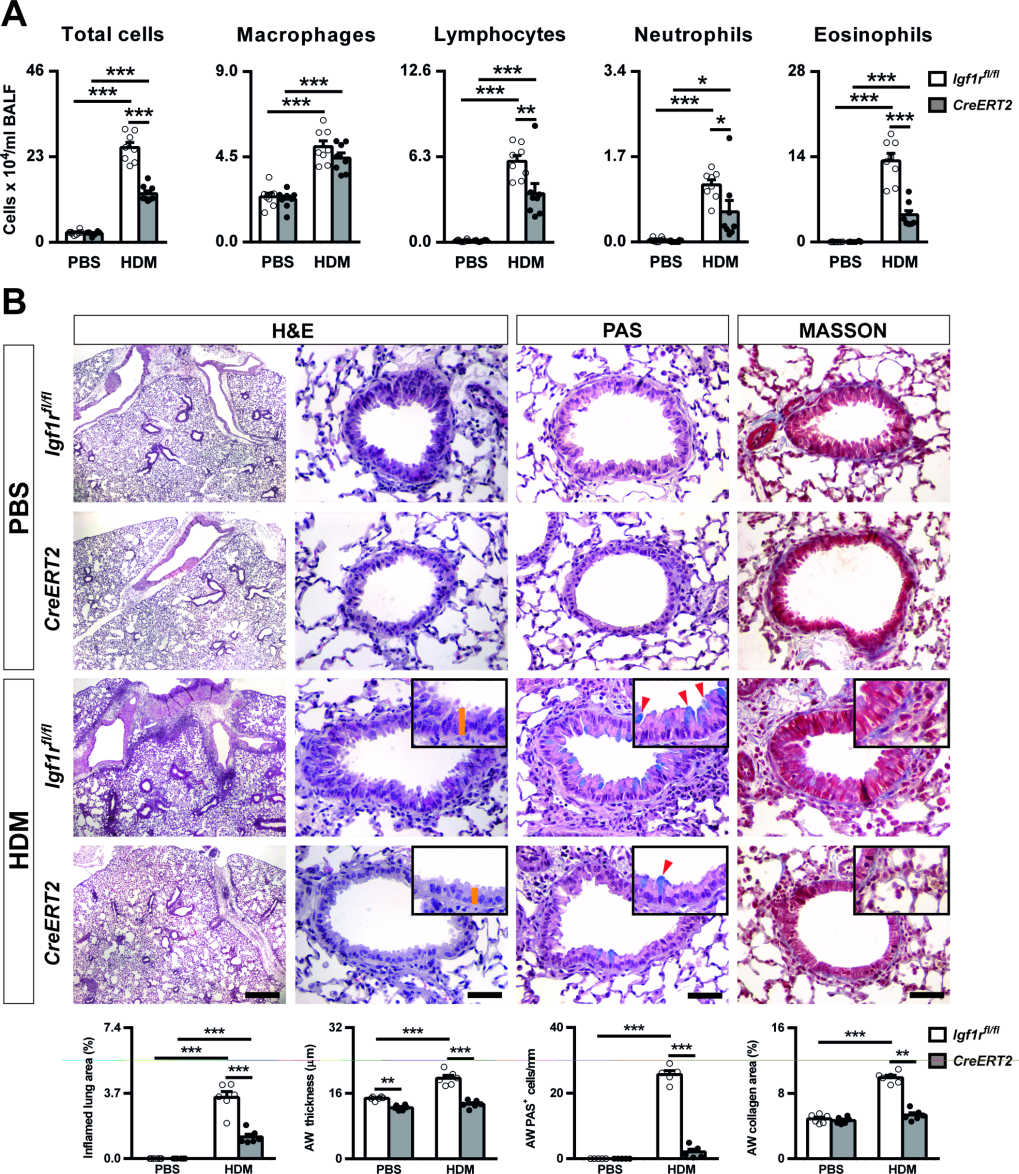
*Igf1r* deficiency decreases airway inflammation and remodeling after HDM exposure. (A) Total and differential cell counts performed on cytospin preparations of BALF (n = 8 animals per group) and (B) representative images of proximal airways showing inflamed lung areas (left) (scale bar: 0.5 mm); airway thickness (orange bars in insets) (center left), mucus-producing cells per epithelium length (red arrowheads in insets) (center right) and collagen content (right) (scale bars: 50 μm; n = 5–6 animals per group) in PBS- or HDM-exposed *Igf1r-*deficient vs. *Igf1*^*fl/fl*^ mice. Bottom graphs represent quantification of the abovementioned parameters. Data are expressed as mean ± SEM *p<0.05; **p<0.01; ***p<0.001 (Dunn-Sidak multiple comparison test). H&E, Hematoxilin and eosin; PAS, Periodic Acid Schiff; AW, airway; HDM, house dust mite; PBS, phosphate buffered saline.

Following allergen challenge, *Igf1r*^*fl/fl*^ mice demonstrated a significant increase in total BALF cells. This effect was less pronounced in *Igf1r*-deficient mice. HDM-treated *Igf1r*^*fl/fl*^ and *Igf1r*-depleted mice showed a marked increase in macrophage, lymphocyte, neutrophil and eosinophil numbers in BALF. Notably, *Igf1r*-deficient mice demonstrated a modest decrease in lymphocyte and neutrophil counts along with a pronounced reduction in eosinophil numbers respect to *Igf1r*^*fl/fl*^ mice (Fig 5A).

Inflamed lung area, number of PAS^+^ cells, collagen staining and airway thickness were notably decreased in lungs from HDM-treated *Igf1r*-deficient mice compared to lungs from *Igf1r*^*fl/fl*^ mice. Whereas HDM induced airway thickening in *Igf1r*^*fl/fl*^ mice, this phenomenon was not observed in *Igf1r*-depleted lungs (Fig 5B).

### Preventively-induced *Igf1r* deficiency involves changes in expression of IGF system genes and reduces allergy-related marker levels

**Fig 6:**
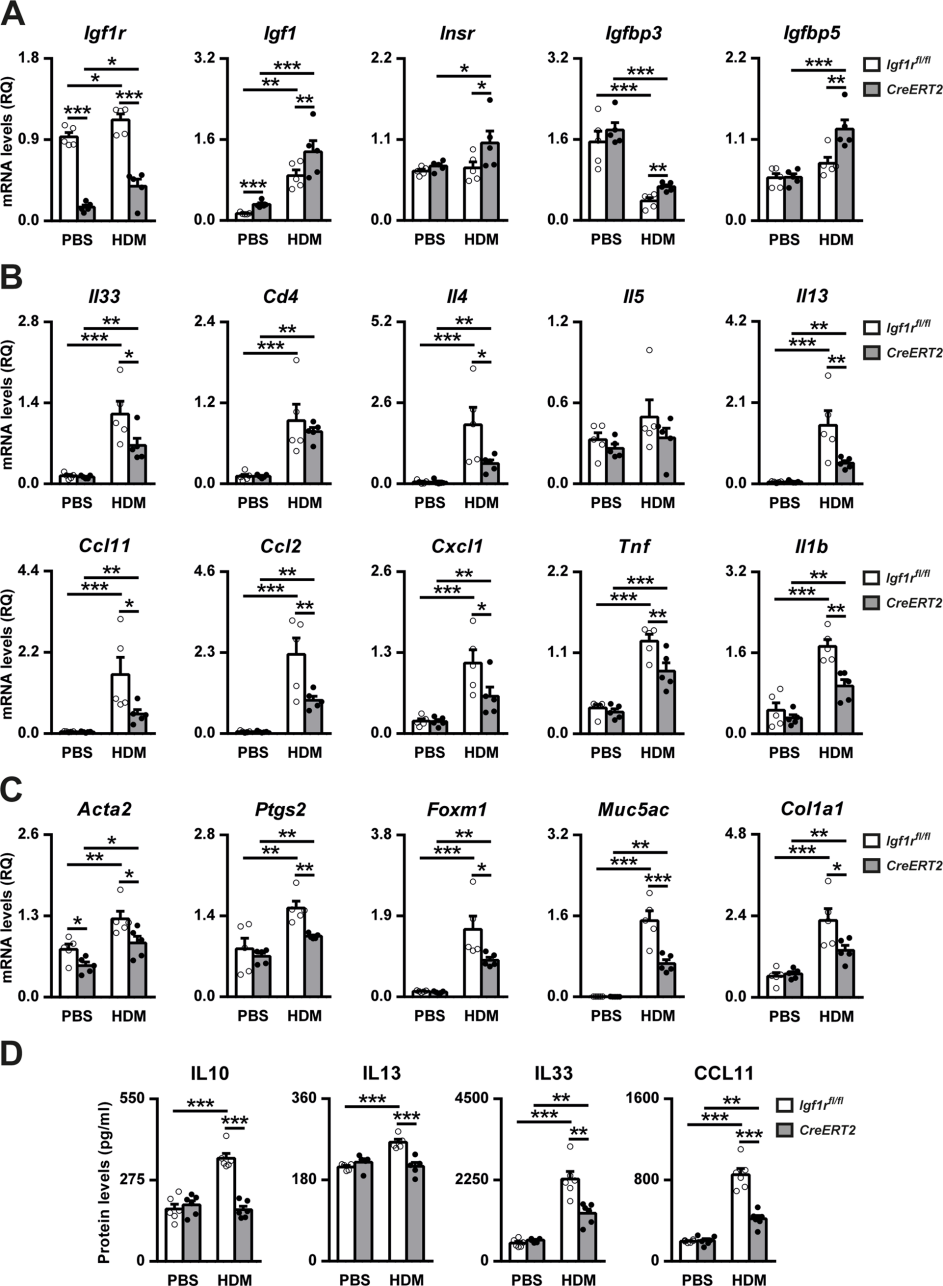
Expression of IGF genes and airway inflammation and remodeling markers in HDM-exposed *Igf1r*-deficient lungs. Lung tissue mRNA expression levels of (A) IGF-system genes (*Igf1r*, *Igf1*, *Insr*, *Igfbp3 and Igfbp5*); (B) dendritic cell activator (*Il33*), T-lymphocyte marker (*Cd4*), Th2 cytokines (*Il4*, *Il5 and Il13*), eosinophil (*Ccl11*), macrophage (*Ccl2*) and neutrophil (*Cxcl1*) chemoattractants and Th1 cytokines (*Tnf and Il1b*); (C) bronchoconstriction (*Acta2 and Ptgs2*), goblet cell hyperplasia (*Foxm1 and Muc5ac*) and collagen deposition (*Col1a1*) markers, and (D) IL10, IL13, IL33 and CCL11 protein levels in lung homogenates in PBS- and HDM-exposed *Igf1r-*deficient and *Igf1*^*fl/fl*^ mice. Data are expressed as mean ± SEM (n = 5–6 animals per group). *p<0.05; **p<0.01; ***p<0.001 (Dunn-Sidak multiple comparison test). HDM, house dust mite; PBS, phosphate buffered saline.

As a complement to the BALF and histopathology analyses, a molecular analysis of IGF system genes and allergic inflammation and remodeling markers was performed. mRNA expression profiles demonstrated an efficient depletion of *Igf1r* levels with *Igf1r*-deficient PBS- and HDM-treated mice showing a reduction of 84% and 67% respectively. HDM treatment increased *Igf1r* expression in *Igf1r*^*fl/fl*^ (17%) and *Igf1r*-depleted (2-fold) lungs. *Igf1* levels were significantly increased in *Igf1r*-deficient PBS- or HDM-treated mice. This effect was augmented in HDM-challenged animals. In addition, HDM treatment increased *Insr*, *Igfbp3* and *Igfbp5* levels in *Igf1r-*deficient lungs. *Igfbp3* expression was decreased in both genotypes (Fig 6A). mRNA levels of all allergic airway inflammation- and remodeling-related markers tested, with the exception of IL5, were strongly induced by HDM and reduced in *Igf1r*-deficient mice, except Cd4 levels (Fig 6B and 6C). The goblet cell hyperplasia marker *Spdef* was also evaluated and found to be significantly decreased in *Igf1r*-deficient HDM-exposed lungs (data not shown, 2-fold reduction). Protein levels of IL13, IL33, and CCL11 were consistent with the mRNA expression profiles. IL10 levels were only increased in HDM-treated *Igf1r*^*fl/fl*^ lungs (Fig 6D).

### Therapeutic *Igf1r*-gene targeting reduces circulating IL33, CCL11 and IgE levels, inflammation and remodeling features

**Fig 7:**
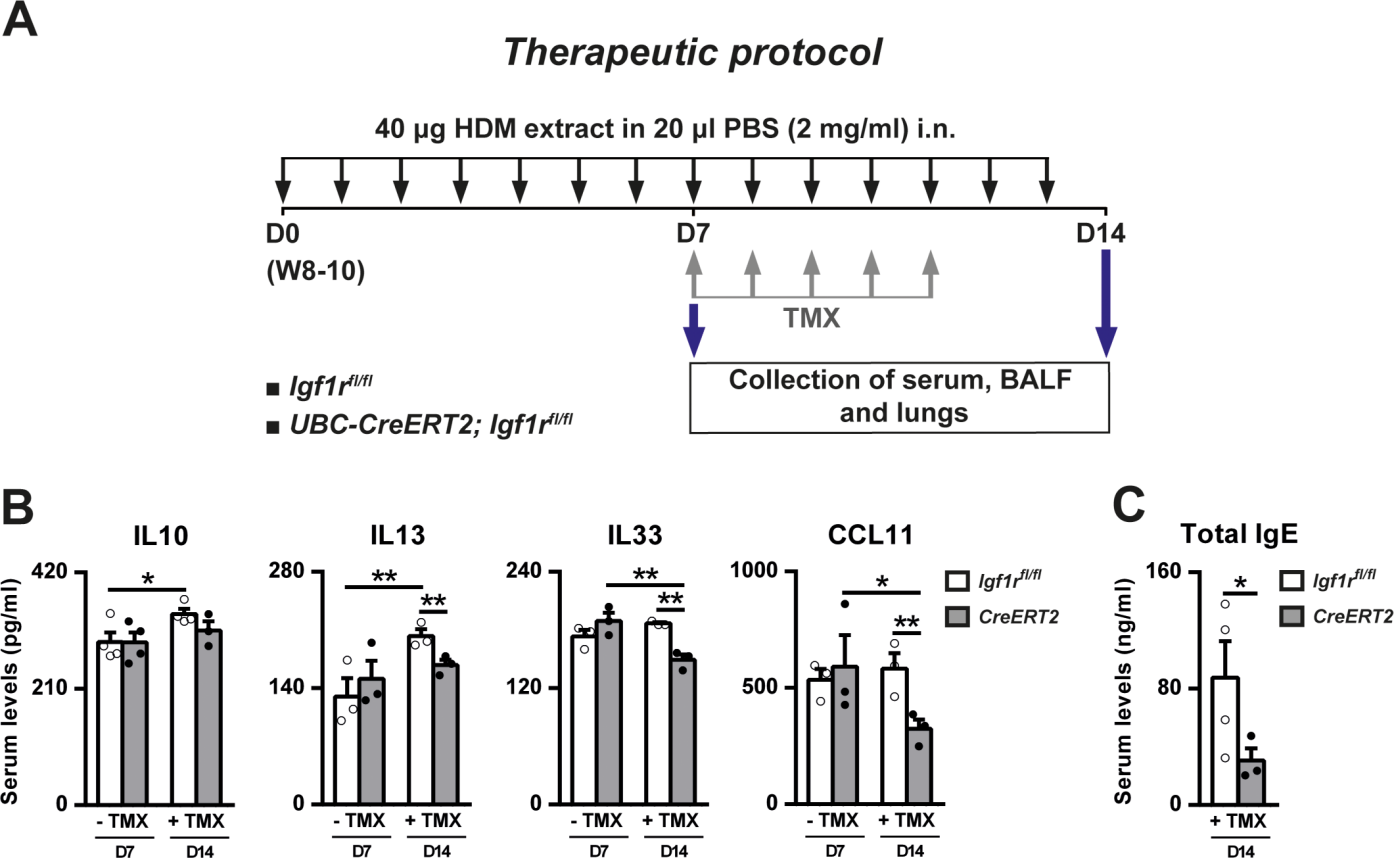
Protocol for therapeutic induction of *Igf1r* deficiency, HDM treatment and circulating levels of allergy-related markers. (A) Eight- to 10-week-old (W8-10) *Igf1r*^*fl/fl*^ and *UBC-CreERT2; Igf1r*^*fl/fl*^ female mice were intranasally challenged with seven (first set of animals non-treated with TMX and sacrificed at D7) or fourteen (second set of animals receiving five consecutive intraperitoneal TMX injections between D7 and D11 to induce *Igf1r* deletion in *UBC-CreERT2; Igf1r*^*fl/fl*^ mice to generate *CreERT2* mice) daily consecutive doses of 40 μg of HDM extract in 20 μl of PBS (2 mg/ml). Serum, BALF and lungs were collected 24 h after the last exposure. (B-C) Serum levels of IL10, IL13, IL33, CCL11 and IgE (n = 3–4 animals per group) in HDM-exposed *CreERT2* vs. *Igf1*^*fl/fl*^ mice after the TMX treatment (D14) (+ TMX). Note that the *CreERT2* term used at D7 (- TMX) refers to *UBC-CreERT2; Igf1r*^*fl/fl*^ mice. Data are expressed as mean ± SEM. *p<0.05; **p<0.01 (Mann-Whitney U and Dunn-Sidak multiple comparison tests). HDM, house dust mite; PBS, phosphate buffered saline; TMX, tamoxifen.

*Igf1r*-deficiency was therapeutically induced in mice to evaluate the resolution of airway inflammation following HDM exposure (Fig 7A). Therapeutic *Igf1r*-gene targeting after TMX administration significantly decreased IL33 and CCL11 serum levels in *CreERT2* compared to non-TMX-treated mice, whereas IL10 and IL13 levels remained unchanged. *Igf1r* depletion significantly reduced IL13, IL33, CCL11 and IgE levels at D14 with respect to *Igf1r*^*fl/fl*^ TMX-treated animals. Interestingly, IL10 and IL13 serum levels increased from D7 to D14 in *Igf1r*^*fl/fl*^ mice (Fig 7B and 7C).

**Fig 8:**
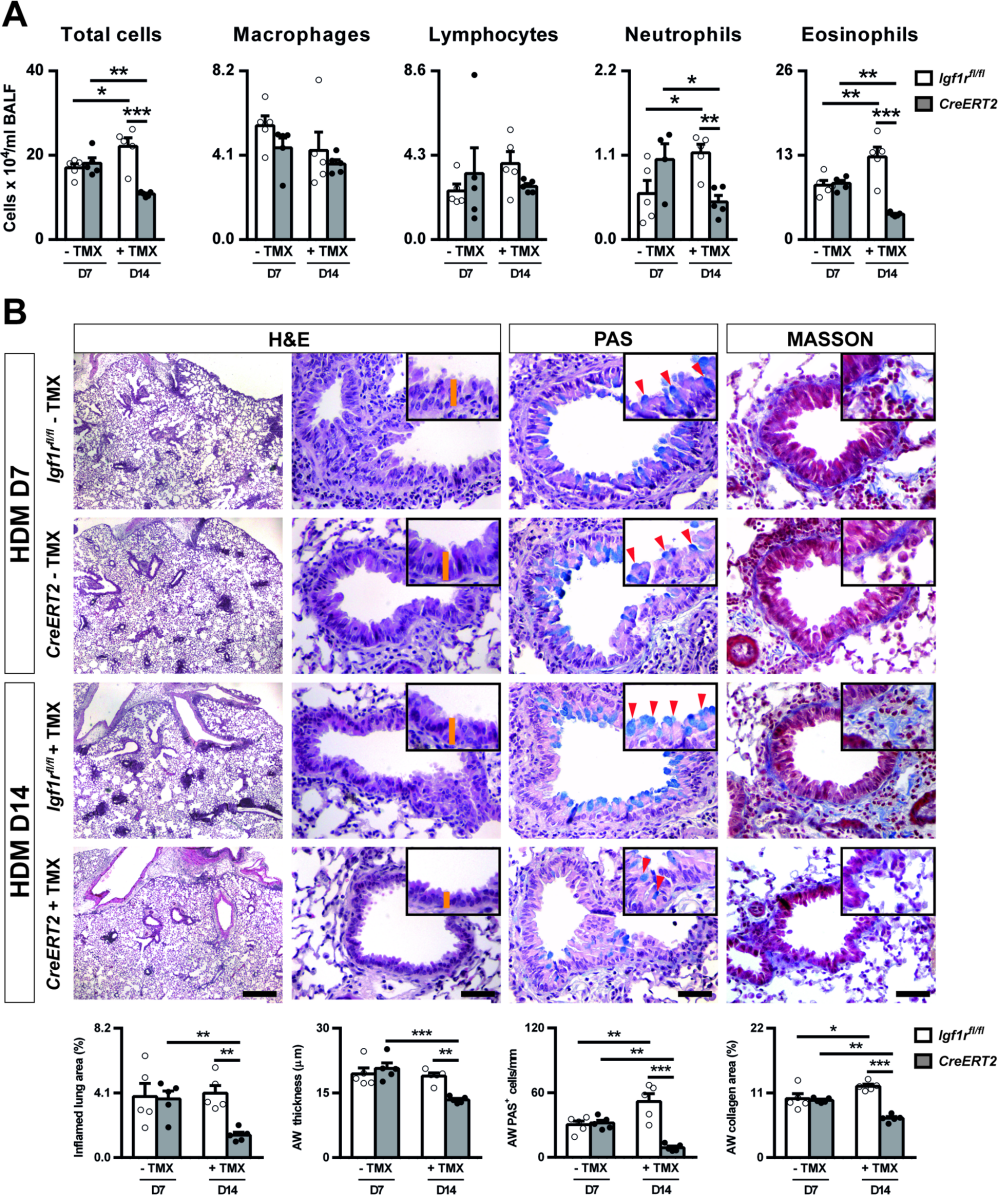
Therapeutic targeting of *Igf1r* reduces airway inflammation and remodeling features after HDM exposure. (A) Total and differential cell counts performed on cytospin preparations of BALF (n = 4–5 animals per group). (B) representative images of proximal airways showing inflamed lung areas (left) (scale bar: 0.5 mm) and airway thickness (orange bars in insets) (center left), mucus-producing cells per epithelium length (red arrowheads in insets) (center right) and collagen content (right) (scale bars: 50 μm) in HDM-exposed *CreERT2* vs. *Igf1*^*fl/fl*^ mice non-treated with TMX (D7) (- TMX) or after the TMX treatment (D14) (+ TMX). Note that the *CreERT2* term used at D7 (- TMX) refers to *UBC-CreERT2; Igf1r*^*fl/fl*^ mice. Bottom graphs represent quantification of the abovementioned parameters (n = 5 animals per group). Data are expressed as mean ± SEM. *p<0.05; **p<0.01; ***p<0.001 (Dunn-Sidak multiple comparison test). H&E, Hematoxilin and eosin; PAS, Periodic Acid Schiff; AW, airway; HDM, house dust mite; PBS, phosphate buffered saline; TMX, tamoxifen.

Total cells in BALF were found to be increased in *Igf1r*^*fl/fl*^ at D14 and substantially reduced in *CreERT2* animals following TMX administration. This effect was attributed primarily to changes in neutrophil and eosinophil counts, since macrophage and lymphocyte numbers remained unchanged (Fig 8A).

Inflamed lung area, airway thickness, PAS^+^ cell numbers and collagen staining were clearly counteracted in *CreERT2* lungs after TMX administration compared to *CreERT2* non-TMX- and *Igf1r*^*fl/fl*^ TMX-treated mice (Fig 8B).

### Therapeutic *Igf1r*-gene targeting diminishes expression of allergic inflammation and remodeling-related markers

**Fig 9:**
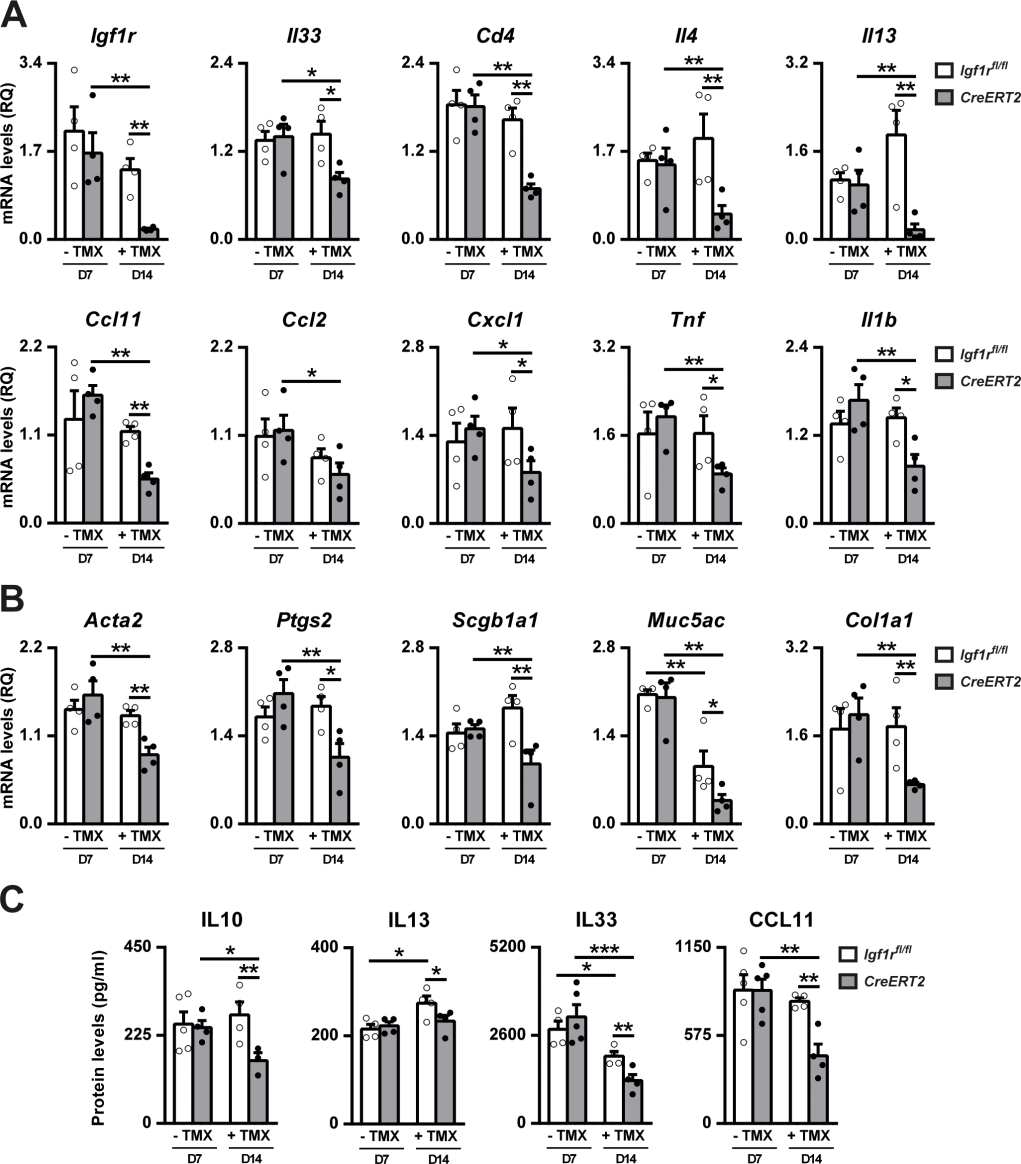
Therapeutic *Igf1r*-gene targeting diminishes expression of airway inflammation and remodeling-related markers after HDM exposure. Lung tissue mRNA expression levels of (A) Insulin-like growth factor 1 receptor (*Igf1r*), dendritic cell activator (*Il33*), T-lymphocyte marker (*Cd4*), Th2 cytokines (*Il4* and *Il13*), eosinophil (*Ccl11*), macrophage (*Ccl2*) and neutrophil (*Cxcl1*) chemoattractants and Th1 cytokines (*Tnf* and *Il1b*) and (B) bronchoconstriction (*Acta2* and *Ptgs2*), goblet cell hyperplasia (*Scgb1a1* and *Muc5ac*) and collagen deposition (*Col1a1*) markers, and (C) IL33, IL10, IL13 and CCL11 protein levels in lung homogenates (n = 3–5 animals per group). Quantifications were performed in HDM-exposed *CreERT2* vs. *Igf1*^*fl/fl*^ mice non-treated with TMX (D7) (- TMX) or after the TMX treatment (D14) (+ TMX). Note that the *CreERT2* term used at D7 (- TMX) refers to *UBC-CreERT2; Igf1r*^*fl/fl*^ mice. Data are expressed as mean ± SEM. *p<0.05; **p<0.01; ***p<0.001 (Dunn-Sidak multiple comparison test). HDM, house dust mite; PBS, phosphate buffered saline; TMX, tamoxifen.

Following TMX administration, *Igf1r* mRNA expression was found to be significantly reduced in *CreERT2* mice compared to *Igf1r*^*fl/fl*^ (85%) and *CreERT2* non-TMX-treated mice (88%). mRNA levels of the allergic airway inflammation- and remodeling-related markers demonstrated a significant reduction in *CreERT2* compared to *Igf1r*^*fl/fl*^ and *CreERT2* non-TMX-treated mice following TMX treatment. *Ccl2* expression was only found reduced in *Igf1r*-deficient mice compared to *CreERT2* TMX-untreated animals (Fig 9A and 9B). Analysis of IL13, IL33 and CCL11 protein levels in lung homogenates supported mRNA data and IL10 levels were found to be significantly decreased in *CreERT2* mice after TMX administration (Fig 9C).

## Discussion

Here we report the progressive changes after acute HDM-induced inflammation in mice. We also demonstrate that preventively-induced *Igf1r*-deficiency ameliorates typical asthmatic features and that therapeutic targeting of *Igf1r* promotes the resolution of HDM-induced inflammation in mice.

Noteworthy, very few studies have reported the acute effects after HDM exposure in mice. In this regard, total and eosinophil counts in BALF and IL13 expression in the lung were found significantly increased one week after repetitive intranasal HDM exposure in mice [15]. Furthermore, total and differential BALF cell counts, peribronchial inflammation and goblet cell hyperplasia were notably increased after 10 consecutive days of intranasal HDM challenge in mice [16]. In the present study HDM exposure in inbred C57BL/6 mice demonstrated a progressive increase in inflammatory cells in BALF, airway remodeling and mRNA expression of allergic airway inflammation and remodeling markers up to D7. Consistent with our results on increased *Il33* expression, it was previously reported that IL33, but not TSLP or IL25, is central to HDM allergic sensitization [17].

Preventive induction of *Igf1r* deficiency in PBS-treated mice led to similar *Igf1r-*depleted expression to that observed in unchallenged mice of similar age [11]. In addition, *Igf1r* and *Igf1* levels increased after HDM exposure. Accordingly, IGF1R and IGF1 expression was found to be increased in BAL cells and bronchial biopsies of asthmatic patients [5,7]. The upregulation of *Igf1* in PBS-treated *Igf1r*-depleted mice or *Igf1* and *Insr* by HDM was possibly due to compensatory effects in response to *Igf1r* deficiency [12]. Regarding increased *Igfbp3* and *Igfbp5* levels in HDM-treated *Igf1r*-deficient mice, exogenous IGFBP3 and IGFBP5 administration blocks the physiological consequences of asthma and enhances epithelial cell adhesion to maintain the epithelial-mesenchymal boundary [9,10,18]. From these data we can conclude that both *Igf1r* and *Igf1* may be important mediators in the establishment of murine asthma, and that *Igfpb3* and *Igfpb5* could play protective roles against HDM-induced allergic inflammation.

Whereas acute HDM treatment caused a clear increase in eosinophil and neutrophil numbers in the bone marrow of *Igf1r*^*fl/fl*^ mice, their levels remained close to basal after preventively-induced *Igf1r*-deficiency. Recent data published by our group showed a selective decrease in circulating eosinophils after chronic HDM exposure and reduced neutrophil numbers after acute-induced lung injury in the same mutant mouse line [11,12]. Thus, IGF1R could have an important role in bone marrow mielopoiesis after HDM-induced allergy.

Following HDM challenge, decreased total and eosinophil counts in BALF and reduced asthmatic features after preventively-induced *Igf1r* deficiency are consistent with published data on IGF1R-deficient mice after chronic HDM exposure [12]. Notably, neutrophil and eosinophil presence in BALF and asthma-related features were counteracted in a similar manner in HDM-treated mice following therapeutic targeting of *Igf1r*.

We demonstrated that IGF1R plays an important role in initiation of the inflammatory process, since IGF1R deficient mice showed reduced *Il1b* and *Tnf* expression [11]. This is consistent with the decreased expression of *Il1b* and *Tnf* either after preventive or therapeutic-induced *Igf1r* deficiency. In this regard, it was reported that both IL1B and TNF are required for allergen-specific Th2 cell activation and for the development of AHR in mice [19,20]. Accordingly, we have reported that IGF1R-deficient mice exhibited no AHR after chronic HDM exposure [12].

Lung inflammation in asthma is typically orchestrated by activation of innate immune cells followed by an exacerbated Th2-biased inflammation and synthesis of allergen-specific IgE antibody, which initiates the release of inflammatory mediators from immune cells [21,22]. In this line, following HDM exposure, *Igf1r*^*fl/fl*^ mice demonstrated increased levels of serum total IgE that were counteracted upon both the preventive and therapeutic strategies. Accordingly, elevated levels of serum total IgE have been reported in HDM-challenged mice and in patients with allergic asthma [12,23].

Upon allergen exposure, IL33 is mainly released from the airway epithelium to participate in the induction of Th2 immunity and is important for the establishment and maintenance of allergic response [24]. IL33 levels were significantly reduced in serum and lungs after therapeutic-induced *Igf1r* deficiency but only in lungs following preventive-induced deficiency. Accordingly, increased expression of IL33 in the airway epithelium and serum of asthmatic patients was correlated with disease severity [25,26]. Additional reports have shown that IL33 exacerbates murine allergic bronchoconstriction and that resolution of allergic airway inflammation and AHR is dependent upon disruption of IL33 signaling in mice [27,28]. Notably, lung epithelial-specific *Igf1r* deficiency in mice caused delayed differentiation of the airway epithelium, a major source of IL33 [29,30]. IGF1R*-*deficient lungs showed a reduced proportion of club cells in distal airways after chronic HDM exposure [12] and therefore this phenomenon could be manifested as a reduced IL33 release from the airway epithelium by HDM.

Here we report attenuated increase in IL13 levels in serum and lungs following preventive or therapeutic induction of *Igf1r* deficiency. Since IL13 levels are increased in serum of asthmatic patients, it is considered a biomarker of disease severity [31]. Blockade of IL13 activity in mice after HDM sensitization reduces eosinophilia in BALF, peribronchial collagen, and goblet cell hyperplasia [32]. These findings are in accordance with results presented in this study and in a recent publication from our group in which IGF1R-deficient mice also showed unaltered IL13 levels after chronic HDM exposure [12].

Following HDM treatment IL10 levels were found to be decreased after both the preventive and therapeutic strategies. Even though IL10 is a regulatory cytokine with immunosuppressive and anti-inflammatory properties, its role in asthma remains unclear. IL10 is necessary for the expression of AHR after allergic sensitization in mice and its levels in serum were reported differently altered in asthmatic patients [26,33,34].

CCL11 is also a potential diagnostic marker for asthma since it is significantly increased in serum of asthmatic patients [35]. We found reduced CCL11 levels in serum and lungs after both the preventive and therapeutic approaches. Notably, enhanced expression of CCL11 in the bronchial epithelium of asthmatic patients was found to be associated with the development of AHR [36]. After allergen exposure, the eosinophil chemoattractant CCL11 is released by the airway epithelium in response to cytokines such as IL4, IL13 and TNF [37]. In the present study, reduced IL13, *Il4* and *Tnf* levels in HDM-treated mice after preventive- or therapeutic-induced *Igf1r* deficiency supported depleted CCL11 levels. Of note, club cell-derived CCL11 is crucial for the accumulation of eosinophils during allergic lung inflammation [38].

The reduced number in PAS^+^ cells was validated by decreased expression of the goblet cell hyperplasia markers *Foxm1*, *Spdef* and *Muc5ac* following preventive induction of *Igf1r* deficiency and by decreased expression of *Scgb1a1* and *Muc5ac* after the therapeutic approach. After allergen stimulation FOXM1 induces differentiation of club cells into goblet cells through transcriptional activation of SPDEF. Then, increased MUC5AC expression by SPDEF in goblet cells contributes to goblet cell hyperplasia and mucus hyperproduction [39]. In accordance, blockade of FOXM1 activity in mice after HDM exposure led to reduced goblet cell hyperplasia and decreased number of eosinophils in BALF [39,40]. Furthermore, we recently demonstrated that lung epithelial-specific *Igf1r* deficiency in mice and chronically HDM-challenged IGF1R-depleted mice showed delayed club cell differentiation which could result in decreased goblet cell hyperplasia and mucus production [12,29].

**Fig 10:**
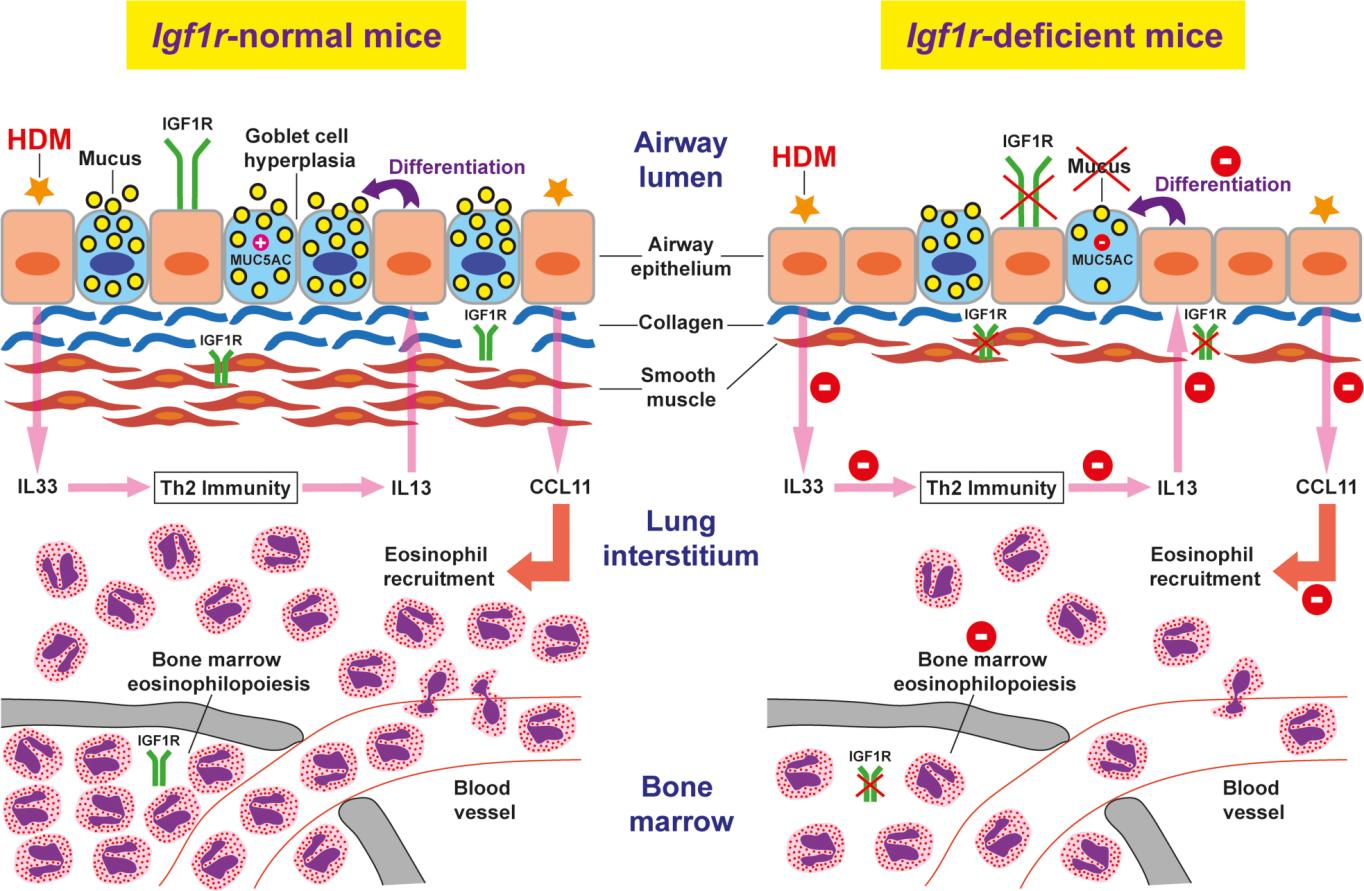
Proposed mechanism for reduced susceptibility to airway inflammation in *Igf1r*-deficient mice upon HDM-challenge. Following HDM exposure *Igf1r* deficiency counteracts collagen deposition, smooth muscle thickening and mucus secretion. The airway epithelium is known to be a major source of IL33 and its delayed differentiation by *Igf1r* deficiency could diminish IL33 levels after HDM treatment, reducing the induction of Th2 immunity and particularly IL13 expression. After HDM exposure, IL13 normally stimulates goblet cell differentiation in the airway epithelium which leads to goblet cell hyperplasia and mucus hyperproduction in addition to triggering the release of CCL11. Delayed differentiation of the airway epithelium caused by *Igf1r* deficiency together with diminished IL13 levels may inhibit differentiation of goblet cells and CCL11 production, reducing mucus secretion and eosinophil recruitment to the lung. Additionally, decreased eosinophilopoiesis in bone marrow of *Igf1r* deficient mice can also substantially contribute to reduced eosinophil presence in the lung. The proposed mechanism illustrated in this figure is supported by results from the present study and additional reports [12,20,28,29,36,38].

Both *Acta2* (α-SMA) and *Ptgs2* (COX2) levels were found to be decreased following preventive and therapeutic approaches. Accordingly, airway smooth muscle thickness was substantially reduced in IGF1R-deficient mice after chronic HDM exposure [12] and pharmacological COX2 inhibition after allergen challenge in mice reduced inflammatory cells in BALF [41]. Moreover, decreased *Col1a1* expression following the preventive and therapeutic approaches supported the reduced collagen deposition around the airways. The proposed mechanism regarding the reduced susceptibility to allergic airway inflammation in *Igf1r*-deficient mice upon HDM-challenge is summarized in Fig 10.

Although the short-term therapeutically-induced and generalized *Igf1r* deficiency presented in this report efficiently resolves established allergic airway inflammation, TMX-mediated *Igf1r* deletion in mice may occur with different degrees of mosaicism in different cell types. Thus, *Igf1r* generalized deficiency cannot be used to deduce in which cells IGF1R signaling is crucial for promoting allergic airway inflammation. Furthermore, the variability of intranasal administration of HDM and the effect of the genetic background on phenotypic variations should also be considered as constraints to this report.

Here we demonstrate that therapeutic targeted deletion of *Igf1r* resolves allergic airway inflammation in response to HDM. These results reinforce our previous findings on the role of IGF1R in allergy, placing it as a potential candidate to develop novel clinical trials focused on the study of systemic IGF1R inhibitors that could be more efficient in counteracting the asthmatic response at different levels.

## Supporting information

S1 Table Primer sets used for qRT-PCR. (PDF)
