# Supplementary material for: Characterization of the acute inflammatory profile and resolution of airway inflammation after Igf1r-gene targeting in a murine model of HDM-induced asthma
Source: PLoS One. 2017 Dec 22;12(12):e0190159. doi: 10.1371/journal.pone.0190159 (PMC5741234; doi:10.1371/journal.pone.0190159)
Supplement: S1 Table — (PDF) [file pone.0190159.s001.pdf]

# *Supporting information for*

## Characterization of the acute inflammatory profile and resolution of airway inflammation after *Igf1r*-gene targeting in a murine model of HDM-induced asthma

**Sergio Piñeiro-Hermida[1], Elvira Alfaro-Arnedo[1], Joshua A. Gregory[2#], Raquel Torrens[1], Carlos Ruíz-Martínez[3], Mikael Adner[2], Icíar P. López[1] and José G. Pichel[1*]**

[1]Lung Cancer and Respiratory Diseases Unit, Centro de Investigación Biomédica de la Rioja (CIBIR), Fundación Rioja Salud, Logroño, Spain. [2]Unit of Experimental Asthma and Allergy Research, Karolinska Institutet, Institute of Environmental Medicine (IMM), Stockholm, Sweden. [3]Pneumology Service, Hospital San Pedro, Logroño, Spain.

[#]Current affiliation: Adlego Biomedical AB, Uppsala, Sweden.

*José G. Pichel, corresponding author.

**Files included:**

**S1 Table**. Primer sets used for qRT-PCR.

**S1 Table.** Primer sets used for qRT-PCR.

| Gene | Accession No. | Forward primer (5´-3´) | Reverse primer (5´-3´) |
|---|---|---|---|
| ***Acta2*** | NM_007392.3 | AATGGCTCTGGGCTCTGTAA | CTCTTGCTCTGGGCTTCATC |
| ***Ccl2*** | NM_011333.3 | CACCAGCCAACTCTCACTGA | CGTTAACTGCATCTGGCTGA |
| ***Ccl5*** | NM_013653.3 | CCAACCCAGAGAAGAAGTGG | AGCAAGCAATGACAGGGAAG |
| ***Ccl11*** | NM_011330.3 | GAGAGCCTACAGAGCCCAGA | ACCGTGAGCAGCAGGAATAG |
| ***Cd4*** | NM_013488.2 | ATGTGGAAGGCAGAGAAGGA | TGGGGTATCTTGAGGGTGAG |
| ***Col1a1*** | NM_007742.4 | CGGAGAAGAAGGAAAACGAG | CAGGGAAACCACGGCTAC |
| ***Cxcl1*** | NM_008176.3 | ATCCAGAGCTTGAAGGTGTTG | GTCTGTCTTCTTTCTCCGTTACTT |
| ***Foxm1*** | NM_008021.4 | CCTGCTTACTGCCCTTTCCT | CACACCCATCTCCCTACACC |
| ***Igfbp3*** | NM_008343.2 | GCCCTCTGCCTTCTTGATTT | TCACTCGGTTATGGGTTTCC |
| ***Igfbp5*** | NM_010518.2 | GATGAGACAGGAATCCGAACAAG | AATCCT TTGCGGTCACAGTTG |
| ***Igf1*** | NM_010512 | CAGAAGCGATGGGGAAAAT | GTGAAGGTGAGCAAGCAGAG |
| ***Igf1r*** | NM_010513 | ATGGCTTCGTTATCCACGAC | AATGGCGGATCTTCACGTAG |
| ***Il1b*** | NM_008361.3 | GCAACTGTTCCTGAACTCAACT | ATCTTTTGGGGTCCGTCAACT |
| ***Il4*** | NM_021283.2 | CCTCACAGCAACGAAGAACA | CGAAAAGCCCGAAAGAGTC |
| ***Il5*** | NM_010558.1 | GAAGTGCTGGAGATGGAACC | GGATGAGGGGGAGGGAGTAT |
| ***Il10*** | NM_010548.2 | GCACTACCAAAGCCACAAGG | TAAGAGCAGGCAGCATAGCA |
| ***Il13*** | NM_008355.3 | GCCTCCCCGATACCAAAAT | CTTCCTCCTCAACCCTCCTC |
| ***Il25*** | NM_080729.3 | AAGCCCTCCAAAGCCCTAC | TCTCCCCAAGTCCTCCATC |
| ***Il33*** | NM_133775.2 | GCCTTGCTCTTTCCTTTTCTC | TCGGTTGTTTTCTTGTTTTGC |
| ***Insr*** | NM_010568.2 | TCCTGAAGGAGCTGGAGGAGT | CTTTCGGGATGGCCTGG |
| ***Muc5ac*** | NM_010844.1 | CACACACAACCACTCAACCA | TCTCTCTCCGCTCCTCTCAA |
| ***Ptgs2*** | NM_011198.4 | GGAGGCGAAGTGGGTTTTA | TGATGGTGGCTGTTTTGGTA |
| ***Rn18s*** | NR_003278.3 | ATGCTCTTAGCTGAGTGTCCCG | ATTCCTAGCTGCGGTATCCAGG |
| ***Scgb1a1*** | NM_011681 | ATGAAGATCGCCATCACAATCAC | GGATGCCACATAACCAGACTCT |
| ***Spdef*** | NM_013891.4 | GGCCAGCCATGAACTATGAT | GGTAGACAAGGCGCTGAGAG |
| ***Tnf*** | NM_013693.3 | GCCTCTTCTCATTCCTGCTTG | CTGATGAGAGGGAGGCCATT |
| ***Tslp*** | NM_021367.2 | AAATGGGAAATGAGCAATAGAC | GCAGGGGAGGTGAGAAAGAC |
